# Relationship between traditional Chinese medicine constitutional types with chemotherapy-induced nausea and vomiting in patients with breast cancer: an observational study

**DOI:** 10.1186/s12906-016-1415-3

**Published:** 2016-11-09

**Authors:** Yi Liu, Ting Pan, Wenjing Zou, Ye Sun, Yun Cai, Rui Wang, Pingping Han, Zhe Zhang, Qunying He, Feng Ye

**Affiliations:** 1 Department of Traditional Chinese Medicine, The First Affiliated Hospital of Xi’an Jiaotong University, Xi’an, 710061 China; 2 Department of Geriatrics, The Fifth Hospital of Xi’an, Xi’an, 710082 China; 3 Department of Infectious Diseases, The First Affiliated Hospital of Xi’an Jiaotong University, Xi’an, 710061 China

**Keywords:** Traditional Chinese medicine, Constitution, Breast cancer, Chemotherapy, Nausea and vomiting

## Abstract

**Background:**

The theory of traditional Chinese medicine (TCM) constitution involves genetic characteristics, psychological factors, organ functions, and many other aspects. Studies have shown that TCM constitution is associated with HLA polymorphisms and has a genetic basis. A large number of Chinese studies have suggested that the clinical evolution of breast cancer may differ among patients with different TCM constitutions. In addition, patients with breast cancer and different TCM constitutions may have different degrees of myelosuppression after chemotherapy. Some studies have revealed that some constitutions may become predictive factors for death and morbidity of some diseases. The study was to investigate the risk factors among TCM constitutions for chemotherapy-induced nausea and vomiting (CINV) in patients with primary breast cancer undergoing chemotherapy.

**Methods:**

From September 2008 to January 2014, 612 patients who underwent surgery and chemotherapy for breast cancer in three hospitals in Xi’an, Shanxi province, underwent TCM constitution assessment using the Nine Basic Constitutions in Chinese Medicine Questionnaire before chemotherapy. CINV was monitored during treatments. Patients were asked to complete the Functional Living Index-Emesis (FLIE) questionnaire. The most severe CINV grade during chemotherapy was recorded according to the WHO standard. The relationships between TCM constitutions, CINV, and clinical and pathological characteristics of the cancers were assessed.

**Results:**

There were no differences in the incidence of CINV among breast cancer patients receiving different chemotherapy regimens, and among patients with different TCM constitutions. The wetness-heat score was an independent risk factor for severe CINV (grade III-IV) (OR = 1.012, 95 % CI: 1.007–1.021, *P* < 0.001). In-depth analyses of the wetness-heat constitution showed that bitter taste/smelly mouth was an independent risk factor for severe CINV (OR = 1.209, 95 % CI: 1.035–1.412, *P* = 0.017), as well as progesterone receptor-positive cancer (OR = 1.429, 95 % CI: 1.030–1.981, *P* = 0.032). Vomiting history was a protective factor against CINV (OR = 0.548, 95 % CI: 0.353–0.849, *P* = 0.007).

**Conclusion:**

Risk of grade III-IV nausea and vomiting was higher in breast cancer patients with TCM constitution of wetness-heat, especially bitter taste or smelly mouth.

## Background

Breast cancer is the most common cancer in women [1]. Endocrine therapy and targeted therapy are becoming increasingly important in the treatment of breast cancer [2], and gene expression profiles are being increasingly used to guide the selection of therapies. However, chemotherapy still plays an important role in breast cancer treatment [2] and its numerous side effects still require specific care. Chemotherapy-induced nausea and vomiting (CINV) is still the most common and one of the most distressing adverse reactions of chemotherapy. Since female gender itself is an independent risk factor for CINV [3], CINV in women with breast cancer might be even more common and severe compared with other cancers. Indeed, 77.3 % of patients with breast cancer experience nausea and 50 % experience vomiting [4], severely affecting the quality of life and requiring anti-emetic treatments [5].

Many risk factors are associated with CINV, among which the emetogenic force of the chemotherapy regimen is the most important factor for severe CINV. However, CINV severity may significantly differ between individuals even if the same chemotherapy regimen is used, and risk factors such as ethnic group, psychological factors, mental preparation for nausea and vomiting, organ status, young age, female gender, prior CINV history, history of morning and motion sickness, and low alcohol use are associated with CINV [6–9]. These studies revealed that for patients receiving anthracyclines, the risk of severe CINV in Asian patients was significantly higher than in non-Asian patients [6].

The theory of traditional Chinese medicine (TCM) constitution involves genetic characteristics, psychological factors, organ functions, and many other aspects [10]. Constitution refers to congenital and acquired inherent characteristics that are comprehensive and relatively stable in morphological structures, physiological functions, and psychological status in the process of human life. Studies have shown that TCM constitution is associated with HLA polymorphisms [11] and has a genetic basis [12]. Therefore, TCM constitutions determine the individual’s specificity [13], which often determines his/her susceptibility to certain pathogenic factors as well as different responses to drugs [9]. According to the theory of TCM constitution, there are nine kinds of constitutions including one normal constitution (gentleness), and eight pathological constitutions (yang-deficiency, yin-deficiency, qi-deficiency, phlegm-wetness, wetness-heat, qi-depression, blood-stasis, and special diathesis).

A large number of Chinese studies have suggested that the characteristics and clinical evolution of breast cancer may differ in patients with different TCM constitutions [14–16], and TCM constitution influences the incidence, development, and prognosis of breast cancer [14]. In addition, patients with breast cancer and different TCM constitutions may have different degrees of myelosuppression after chemotherapy [17]. Some studies have revealed that some constitutions may become predictive factors for death and morbidity of some diseases [18].

Therefore, the present study aimed to investigate the association between TCM constitutional types and CINV in patients with breast cancer. Results could help identifying risk factors for severe CINV and help clinicians to manage CINV more effectively in these patients.

## Methods

### Patients

Patients who were postoperatively diagnosed with breast cancer upon histological examination of the surgical specimen at The First Hospital of XI’an Jiaotong University, the Shaanxi General Hospital of Chinese Armed Police Force (CAPF), and the Shaanxi Province Hospital of traditional Chinese medicine between September 2008 and January 2014 were screened for eligibility (*n* = 1745).

**Fig. 1:**
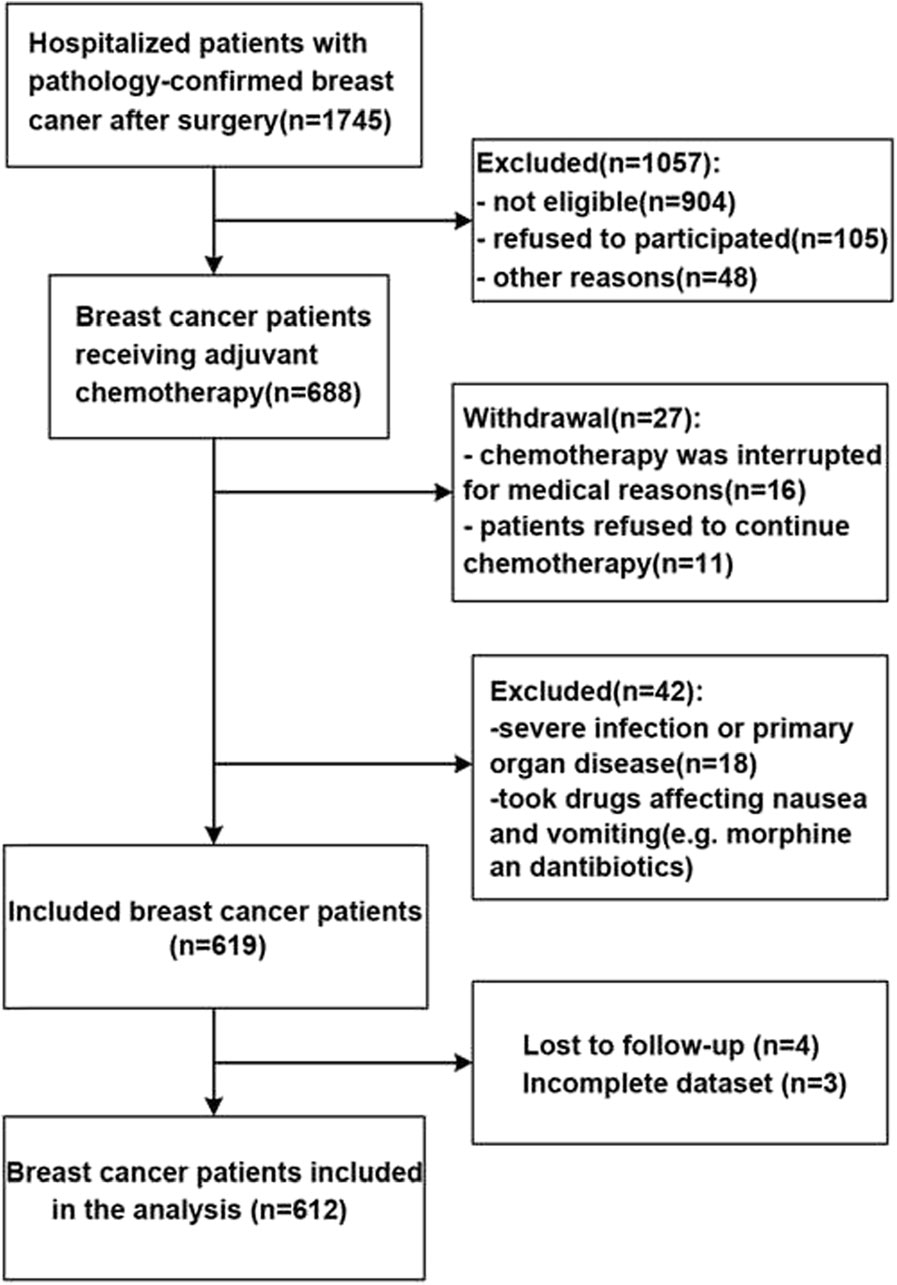
Patients’ flowchart

Inclusion criteria were: 1) histologically confirmed breast cancer; 2) underwent surgery; and 3) Karnofsky performance status (KPS) score ≥60. Exclusion criteria were: 1) received radiotherapy and/or chemotherapy within one year before surgery; 2) severe infection, severe dyscrasia, severe primary diseases (such as cardiovascular disease, cerebrovascular disease, or disease of the liver, kidney, hematopoietic system, endocrine system, mental disease, or sarcopenia); 3) any diagnosis of gastrointestinal disease; 4) nausea or vomiting before chemotherapy; or 5) taking drugs that might affect the incidence of nausea and vomiting (such as antiviral drugs, antibiotics, or morphine). Patients were allowed to withdraw from chemotherapy for medical reasons or based on patients’ will, but those who experienced CINV before withdrawal were included in the analyses. Eventually, 619 patients were included in the study, and 612 were analyzed (four patients were lost to follow-up, and three had incomplete dataset) (Fig. 1).

### Ethics, consent and permissions

The study was involved human participants and approved by the ethics committee of the First Affiliated Hospital of Xi’an Jiaotong University and then was subsequently passed by the ethics committee of the other two participating hospitals.

### Data collection

Before chemotherapy, the general characteristics of the patients were collected including age, education level, and family history of breast cancer. History of nausea and vomiting was defined as any persistent event that affected their lives and/or required medical interventions. Before chemotherapy, the therapy-related clinical characteristics of the patients were collected including TNM staging, pathological type of the tumor, KPS score, chemotherapy regimen, estrogen receptors (ER) status, progesterone receptor (PR) status, human epidermal growth factor receptor-2 (HER2) status, p53 protein expression, p21 protein expression, and PCNA expression. To determine the expression status of different proteins, positive cells were counted in 10 random and non-overlapping fields at high magnification and each field had to have at least 100 tumor cells. Positive ER and PR statuses were based on the ≥1 % threshold of the ASCO-CAP [19]. HER2, p53, p21, and PCNA positivity was based on a >10 % threshold.

### Assessment and treatment of nausea and vomiting

Three to five days before chemotherapy, the patients were instructed by specialized physicians how to use the visual analogue scale (VAS) for nausea [20]. The VAS was a 100-mm ruler divided into 10 intervals from left to right, where the left end was 0 (no nausea) and the right end was 10 (the most imaginable violent nausea). After each course of chemotherapy was started, the patients were required to keep a 120-h log of the occurrence of nausea and vomiting, which required to record any kind of nausea and/or vomiting including frequency, symptoms, and measures taken. The logs were examined daily by specialized physicians. Patients were asked to complete the FLIE questionnaire by applying the VAS for nausea severity on the 6^th^ day after chemotherapy. A FLIE total score >108 points meant that the nausea and vomiting were tolerable and had no effect on the daily life [21]. If the patients were not hospitalized on the 6^th^ day after chemotherapy, they were followed up by phone and were asked to complete the log, hereby ensuring the validity and reliability of data collection. The logs were collected at each cycle of chemotherapy.

During and after chemotherapy, CINV was graded for each patient according to the WHO standard, and the highest grade of each patient was taken for statistical analyses. Grade 0: no nausea and vomiting; Grade I: only nausea; Grade II: temporary and tolerable vomiting; Grade III: intolerable vomiting that required treatments; and Grade IV: uncontrollable vomiting. Grades 0-II were classified as mild nausea and vomiting, while Grades III-IV were classified as severe nausea and vomiting.

All patients received prophylactic antiemetics, but were not receiving preventive non-pharmacological interventions such as behavioral therapy, acupuncture, and TCM therapy. The beginning of each chemotherapy cycle was considered as “day 1” for conducting standard antiemetic prevention: ondansetron 8 mg IV on day 1 (15 min before, and 4 and 8 h after chemotherapy) and on days 2–5; and dexamethasone injection 10 mg IV on days 1–3. Patients with anticipatory CINV received lorazepam.

Patients with grade ≥ III nausea and vomiting immediately received antiemetic drugs or participated in non-pharmacological interventions. Before each cycle of chemotherapy, routine blood tests, liver function, kidney function, and ECG were checked, and chemotherapy-related side effects were assessed according to the WHO acute and subacute toxicity grading of anticancer drugs. Patients with side effects received symptomatic treatment, and the continuation of chemotherapy was determined by the attending physician according to the severity of the side effects.

### Chemotherapy regimens

Chemotherapy regimens were selected according to the NCCN guidelines (http://www.nccn.org/) issued from 2008 to 2013 and according to the financial means of the patients. Each patient received 2–6 cycles of chemotherapy, with a median of 4 cycles. Ultimately, patients receiving any one of the following eight regimens were enrolled: CAF (cyclophosphamide, adriamycin, and fluorouracil), 28-day cycles; CEF (cyclophosphamide, epirubicin, and fluorouracil), 21-day cycles; AC (adriamycin and cyclophosphamide), 21-day cycles; EC (epirubicin and fluorouracil), 21-day cycles; TAC (docetaxel, adriamycin, and cyclophosphamide), 21-day cycles; TEC (docetaxel, epirubicin, and cyclophosphamide), 21-day cycles; TA (pacilitaxel and adriamycin), 21-day cycles; and TE (docetaxel and epirubicin), 21-day cycles.

### TCM constitutional types

Before chemotherapy, the patients’ TCM constitutional types were classified and quantified according to the Nine Basic Constitutions Classification and Criteria in Chinese Medicine [12, 22] issued by the China Association of Chinese Medicine. The Constitution in Chinese Medicine Questionnaire includes 60 items (please see the Appendix), in which items of nine subscales (each subscale consists of 7–8 items) are interspersed irregularly. For each item, an appropriate answer was selected from a Likert scale (“no”, “occasionally”, “sometimes”, “often”, and “always”, scored 1–5). For each subscale, the original score was first calculated (original score = sum of the scores for each item) and converted into the derived score [(actual score-the possible lowest score of the subscale)/(possible highest score- possible lowest score)×100]. The derived score of each subscale ranged 0–100 points. Ultimately, the TCM constitution was diagnosed. Gentle constitution referred to the patients with derived scores of the eight deviation constitutions being <30 points, and the derived score of the gentleness constitution being ≥60 points. Deviation constitutions were diagnosed if the score of any constitution subscale was ≥40 points. Patients with a derived score of a deviation constitution between 30 and 40 points were referred to as “tendency of deviation constitution”. Patients with two or more constitutional types were recorded twice or more. Patients with a “tendency of deviation constitution” were excluded from the statistical analysis because they were not diagnosed with the deviation constitution. Patients diagnosed with “special diathesis” were excluded from the analysis of risk factors of CINV due to the very small number of cases (*n* = 4).

### Statistical analysis

Statistical analyses were performed using SPSS 16.0 (IBM, Armonk, NY, USA). Continuous data are expressed as mean ± standard deviation and were compared using ANOVA with the Tukey’s post hoc test. Categorical data are expressed as frequencies and were analyzed using the Pearson chi-square test. Variables associated with CINV at *P* < 0.10 in univariate analyses were included in the multivariate model. Because of the small number of patients in some constitutions, the relationship between the nine TCM constitutions and CINV was analyzed using logistic regression based on the actual scores of each constitution instead of the diagnoses themselves. Two-sided *P*-values <0.05 were considered statistically significant.

## Results

### Characteristics of the patients

Figure 1 presents the patients’ flowchart. Table 1 presents the characteristics of the patients. Among all patients, 18.6 % had a history of nausea/vomiting. CINV severity was grade 0 in 7.8 % of the patients, grade I-II in 47.5 %, and grade III-IV in 44.7 %. The chemotherapy regimens are presented in Table 2. There were no significant differences in the incidence of CINV between the regimens (*P* = 0.342).

**Table 1:** Characteristics of the patients

Index		*N* (%)
Age (year)	<35	12 (2.0)
	35–60	544 (88.8)
	>61	56 (9.2)
Education level	Junior middle school or above	250 (40.9)
	Below junior middle school	362 (59.1)
Family history of breast cancer	Yes	206 (33.7)
History of vomiting^a^	Yes	114 (18.6)
TNM staging	I	135 (22.1)
	II	331 (54.0)
	III	104 (17.0)
	IV	42 (6.9)
Pathological type	Infiltrating ductal carcinoma	294 (48.0)
	Infiltrating lobular carcinoma	183 (29.9)
	Non-infiltrating intraductal papillary carcinoma	135 (22.1)
KPS score	60–70	242 (39.5)
	70–100	370 (60.5)
Severity of CINV	Grade 0	48 (7.8)
	Grade I-II	291 (47.6)
	Grade III-IV	273 (44.6)

*TNM* tumor-node-metastasis staging, *KPS* Karnofsky performance status, *CINV* chemotherapy-induced nausea and vomiting

^a^History of nausea and vomiting was defined as any persistent event of nausea and vomiting that affected their lives and/or required medical interventions

**Table 2:** Incidence of nausea and vomiting among breast cancer patients after receiving different chemotherapy regimens (%)

Regimens	n (*n* = 612)	CINV [n (%)]	
		Grade 0-II (*n* = 339)	Grade III-IV (*n* = 273)
CAF	59	36 (61.0)	23 (39.0)
CEF	107	59 (55.1)	48 (44.9)
TEC	57	29 (50.9)	28 (49.1)
TE	122	74 (60.7)	48 (39.3)
TAC	61	40 (65.6)	21 (34.4)
TA	51	24 (47.1)	27 (52.9)
AC	40	21 (52.5)	19 (47.5)
EC	115	56 (48.7)	59 (51.3)

Comparison between groups showed that the incidence of nausea and vomiting between the different chemotherapy groups was not statistically significant (chi-square test, *P* = 0.342)

*CINV* chemotherapy-induced nausea and vomiting, *CAF* cyclophosphamide, adriamycin, fluorouracil, *CEF* cyclophosphamide, epirubicin, and fluorouracil, *AC* adriamycin and cyclophosphamide, *EC* epirubicin and fluorouracil, *TAC* docetaxel, adriamycin, and cyclophosphamide, *TEC* docetaxel, epirubicin, and cyclophosphamide, *TA* pacilitaxel and adriamycin, *TE* docetaxel and epirubicin

### TCM constitutional types

A total of 510 constitution cases were analyzed, which was smaller than the total number of patients because it was impossible to achieve a TCM constitutional diagnosis in some patients. Among these 510 cases, there were 77 cases of gentleness constitution (15.0 %), 146 of qi-depression constitution (28.6 %), 83 of qi-deficiency constitution (16.3 %), 66 of yang-deficiency constitution (12.9 %), 58 of yin-deficiency constitution (11.4 %), 34 of blood-stasis constitution (6.7 %), 22 cases of Wetness-heat constitution (4.3 %), 20 cases of phlegm-wetness constitution (3.9 %), and four of special diathesis constitution (0.8 %) (Table 3). There were no differences in the severity of CINV between the different constitution groups (*P* = 0.529).

**Table 3:** Incidence of CINV among breast cancer patients with different TCM constitutions

Constitution type	n (*n* = 510)	CINV [n (%)]	
		Grace 0-II (*n* = 236)	Grade III-IV (*n* = 264)
Gentleness	77	37 (48.1)	40 (51.9)
Qi-deficiency	83	38 (45.8)	45 (54.2)
Yin-deficiency	58	27 (43.8)	21 (36.2)
Wetness-heat	22	8 (36.4)	14 (63.6)
Qi-depression	146	66 (45.2)	80 (54.8)
Yang-deficiency	66	32 (48.5)	34 (51.5)
Blood-stasis	34	18 (52.9)	16 (47.1)
Phlegm-wetness	20	10 (50)	10 (50)
Special diathesis	4	0 (0)	4 (100)

Comparison between groups showed that the incidence of nausea and vomiting between the different TCM constitution groups was not statistically significant (*P* = 0.529)

*CINV* chemotherapy-induced nausea and vomiting

### Risk factors for severe CINV

Table 4 presents the univariate analysis of clinical characteristics in relation to CINV severity. History of vomiting was more frequent in the mild CINV group compared with the severe CINV group (22.7 % vs. 13.6 %, *P* = 0.004). TNM staging distribution was different between the two groups (*P* = 0.03).

**Table 4:** Univariate analysis of clinical characteristics and constitution scores with severe CINV

		CINV [*n* (%)] (*n* = 612)		
		Grade 0-II (*n* = 339)	Grade III-IV (*n* = 273)	*P*
Age		48.5 ± 10.1	48.5 ± 9.7	0.382
Education level	Junior middle school or above	147 (43.4 %)	103 (37.7 %)	0.164
	Below junior middle school	192 (56.6 %)	170 (62.3 %)	
History of breast cancer	Yes	119 (35.1 %)	87 (31.9 %)	0.400
	No	220 (64.9 %)	186 (68.1 %)	
History of vomiting	Yes	77 (22.7 %)	37 (13.6 %)	0.004
	No	262 (77.3 %)	236 (86.4 %)	
KPS score	60–70	262 (77.3 %)	215 (78.7 %)	0.663
	70–100	77 (22.7 %)	58 (21.3 %)	
TNM staging	I	62 (18.3 %)	73 (26.7 %)	0.030
	II	199 (58.7 %)	132 (48.3 %)	
	III	58 (17.1 %)	46 (16.9 %)	
	IV	20 (5.9 %)	22 (8.1 %)	
ER	Positive	102 (30.1 %)	89 (32.6 %)	0.505
	Negative	237 (69.9 %)	184 (57.4 %)	
PR	Positive	145 (42.8 %)	137 (50.2 %)	0.068
	Negative	194 (57.2 %)	136 (49.8 %)	
HER2	Negative	213 (62.8 %)	171 (62.6 %)	0.961
	Positive	126 (37.2 %)	102 (37.4 %)	
P21	Negative	259 (76.4 %)	199 (72.9 %)	0.320
	Positive	80 (23.6 %)	74 (27.1 %)	
P53	Negative	289 (85.3 %)	221 (81.0 %)	0.156
	Positive	50 (14.7 %)	52 (19.0 %)	
PCNA	Negative	267 (78.8 %)	206 (75.5 %)	0.332
	Positive	72 (21.2 %)	67 (24.5 %)	
TCM constitutions	Qi-deficiency	21.3 ± 13.8	22.7 ± 14.4	0.439
	Yin-deficiency	20.3 ± 13.7	21.5 ± 11.5	0.001
	Wetness-heat	14.6 ± 10.5	17.6 ± 12.5	0.012
	Qi-depression	26.3 ± 16.2	29.4 ± 16.0	0.968
	Yang-deficiency	18.8 ± 15.8	20.0 ± 16.4	0.341
	Blood-stasis	19.3 ± 12.1	21.3 ± 12.4	0.453
	Phlegm-wetness	17.3 ± 10.5	18.3 ± 11.5	0.028
	Gentleness	69.0 ± 12.7	68.0 ± 13.7	0.275

*TNM* tumor node metastasis staging, *KPS* Karnofsky performance status, *CINV* chemotherapy-induced nausea and vomiting, *ER* estrogen receptors, *PR* progesterone receptor, *HER2* human epidermal growth factor receptor 2, *PCNA* proliferating cell nuclear antigen

In univariate analyses, Yin-deficiency (*P* = 0.001), wetness-heat (*P* = 0.012), and phlegm-wetness (*P* = 0.028) types were associated with severe CINV (Table 4). Table 5 shows that a history of vomiting was an independent protecting factor against severe CINV (OR = 0.548, 95 % CI: 0.353–0.849, *P* = 0.007), while progesterone receptor positivity (OR = 1.429, 95 % CI: 1.030–1.981, *P* = 0.032) and the wetness-heat score (OR = 1.021, 95 % CI: 1.007–1.021, *P* < 0.001) were risk factors for severe CINV.

**Table 5:** Multivariate analysis of risk factors for severe CINV among breast cancer patients

	*P*	OR	95 % confidence interval	
			Lower limit	Upper limit
History of vomiting	0.007	0.548	0.353	0.849
PR positivity	0.032	1.429	1.030	1.981
Wetness-heat score	<0.001	1.012	1.007	1.021
TNM staging	0.550	1.063	0.870	1.300
Yin-deficiency score	0.376	0.993	0.977	1.009
Phlegm-wetness score	0.593	0.995	0.978	1.013

Logistic regression, forward conditional method

*OR* odds ratio, *PR* progesterone receptor

Table 6 shows the univariate and multivariate analyses of the items of the wetness heat constitution. Compared with patients with mild CINV, patients with severe CINV showed higher scores for greasy feeling/shining face or nose (*P* < 0.001), prone to acne or furuncles (*P* = 0.004), and sticky stool/sense of endless flow (*P* < 0.001). Bitter taste/smelly mouth was an independent risk factor for severe CINV (OR = 1.209, 95 % CI: 1.035–1.412, *P* = 0.017).

**Table 6:** Univariate and multivariate analyses of the six items of the wetness-heat constitution for severe CINV among breast cancer patients

	Original score (*n* = 612)			Multivariate analysis			
	Grade 0-II (*n* = 339)	Grade III-IV (*n* = 273)	*P*	OR	95 % confidence interval		*P*
					Lower limit	Upper limit	
Greasy feeling or shinning face or nose	1.50 ± 0.82	1.67 ± 1.03	<0.001	1.154	0.965	1.381	0.117
Prone to acne or furuncle	1.19 ± 0.52	1.26 ± 0.63	0.004	1.182	0.887	1.574	0.253
Bitter taste or smelly mouth	1.80 ± 1.02	2.05 ± 1.11	0.056	1.209	1.035	1.412	0.017
Sticky stool and sense of endless flow	1.42 ± 0.75	1.56 ± 0.89	<0.001	1.147	0.936	1.405	0.186
Hot flash in urethra and dark urine	1.54 ± 0.77	1.59 ± 0.86	0.051	1.041	0.848	1.277	0.703
Yellow leukorrhea	1.62 ± 0.96	1.68 ± 0.96	0.260	–	–	–	–

Multivariate analysis was performed through logistic regression, forward conditional method

*OR* odds ratio

## Discussion

Over the past 25 years, revolutionary progress has been achieved in preventing CINV. It is now possible to avoid CINV in most patients using the methods recommended by the different available guidelines [2], but complete prevention is still unachievable [23]. Therefore, a better understanding of the risk factors for CINV could help the clinicians in the management of their patients. Therefore, the aim of the present study was to investigate the risk factors among TCM constitutions that are associated with CINV among patients with primary breast cancer undergoing chemotherapy. Results revealed that there was no difference in the incidence of CINV among breast cancer patients receiving different chemotherapy regimens, and among patients with different TCM constitutions. The wetness-heat constitution score was an independent risk factor for severe CINV. In-depth analyses of the wetness-heat constitution showed that bitter taste or smelly mouth was a risk factor for severe CINV. Progesterone receptor positivity was a risk factor for severe CINV. Vomiting history was an independent protective factor against CINV.

In the present study, most patients with breast cancer had a deviation in TCM constitution, mainly qi-depression, qi-deficiency, yang-deficiency, and yin-deficiency constitutions, among which the last three constitutions belong to weakened body resistance [10]. Some studies revealed that breast tumor size <2.0 cm was commonly seen in patients with qi-depression and weakened body resistance [24], which support the present study. Qi-depression is associated with uncomfortable liver and often manifests clinical syndromes such as depression, nervousness, and anxiety [10].

In the present study, CINV severity was classified as mild (grade 0-II) or severe (grade III-IV). Although the incidence of CINV was not different among patients with different TCM constitutional types, multivariate analyses showed that the wetness-heat constitution score was an independent risk factor of CINV. According to the theory of TCM, the formation of the wetness-heat constitution is associated with wet climate or wet living environment, but also with excessive alcohol, sweet or greasy foods, and digestive disorders. Previous studies have suggested that a history of alcohol intake is a risk factor of CINV, suggesting that patients shall probably reduce alcohol consumption, but also shall reduce the intake of greasy and sweet foods during chemotherapy [3]. In the present study, the incidence of CINV among patients diagnosed with wetness-heat constitution was slightly higher than in the other groups. Nevertheless, as the score was an independent risk factor for severe CINV, it could be considered that the risk of vomiting was significantly increased in patients without meeting all the items of the wetness-heat constitution. Therefore, the components of the wetness-heat constitution were further analyzed and the results showed that bitter taste/smelly mouth was an independent risk factor for severe CINV. The use of CINV prophylaxis might more pertinent in these patients.

Previous studies revealed that some clinical characteristics are risk factors for CINV such as history of hyperemesis gravidarum, history of nausea/vomiting, anxiety, expectation of nausea, young age, and tumor-related fatigue [7–9]. At the participating hospitals, chemotherapy regimens with a high emetogenic potential are often used, and anti-emesis prophylaxis is rarely used because of patients’ financial limitations. Therefore, only 48 patients did not suffer from CINV, preventing the assessment of whether a history of nausea/vomiting was a risk factor of CINV. However, compared with patients without a history of nausea/vomiting, a larger proportion of patients with a history of nausea/vomiting had mild CINV. A previous study found that a history of vomiting could be considered as a risk factor of CINV during the 2^nd^ cycle of chemotherapy, but not during the 3^rd^ cycle of chemotherapy [8], suggesting that a history of nausea/vomiting is only of significance at a specific stage of the chemotherapy process. Therefore, in-depth studies are needed to clarify whether a history of nausea/vomiting is a risk factor of CINV.

The present study suggests that patients with PR-positive breast cancer had a higher probability of severe CINV than patients with PR-negative cancer. However, further study is necessary to assess this point. Age was not found to be a risk factor of CINV, which might be because most patients (88.9 %) were 35–60 years old. In addition, according to the TCM, qi-depression is associated with anxiety [10]. However, qi-depression was not found to be a risk factor of CINV in this study. Since TCM constitutions are a group of symptoms, considering TCM constitutions as risk factors may lead to the omission of some useful clinical information. Thus, in subsequent research, the 60 items of the Constitution in Chinese Medicine Questionnaire should be separately considered as risk factors. In addition, patients with the special diathesis constitution were excluded from the analyses because of the very small number of cases (*n* = 4). Nevertheless, it is worth noting that all four cases with special diathesis suffered from severe CINV. According to TCM, the special diathesis constitution is a kind of allergic constitution and it might be possible that this constitution is associated with an adverse reaction to chemotherapy [10].

The present study is not without limitations. Indeed, even if the sample size was large, some subgroups were too small for statistical analysis; and analyses according to the chemotherapy regimens were not possible. In addition, the variety of chemotherapy regimens might have influenced the results since their emetogenic potentials were different. Finally, there could be some bias since hospitalized patients filled the paper form of the FLIE under assistance of medical staff, if needed, while patients at home responded to the FLIE by phone.

## Conclusions

TCM constitutional types are unable to predict the occurrence of severe CINV, while higher wetness-heat score, especially with bitter taste/smelly mouth, is a risk factor of severe CINV. PR positivity was also a risk factor for severe CINV, while a history of vomiting was a protection factor against severe CINV.

## Appendix

### The Constitution in Chinese Medicine Questionnaire

1. Not at all
2. few
3. sometimes
4. often
5. always

1. Are you full of energy? 1,2,3,4,5
2. Do you easily tired? 1,2,3,4,5
3. Are you susceptible to shortness of breath? 1,2,3,4,5
4. Are you susceptible to (fast heartbeat)? 1,2,3,4,5
5. Are you susceptible to feel dizzy? Or when you stand up, you feel dizzy? 1,2,3,4,5
6. Would you like to be quiet and lazy to talk? 1,2,3,4,5
7. Do you have a low voice? 1,2,3,4,5
8. Are you easy to forget things? 1,2,3,4,5
9. Do you feel bored and depressed? 1,2,3,4,5
10. Are you nervous and anxious? 1,2,3,4,5
11. Do you always feel melancholy? 1,2,3,4,5
12. Are you susceptible to feel scared or frightened? 1,2,3,4,5
13. Do you feel chest pain? 1,2,3,4,5
14. Do you feel chest tightness (especially in rainy and humid weather)? 1,2,3,4,5
15. Are you susceptible to sigh without special reason? 1,2,3,4,5
16. Do you feel your body too heavy to move? 1,2,3,4,5
17. Do you feel your hands and feet are hot? 1,2,3,4,5
18. Do you feel your hands and feet are cold? 1,2,3,4,5
19. Do you feel a cold waist ? 1,2,3,4,5
20. Do you feel cold, and wear more than others? 1,2,3,4,5
21. Do you feel the hot of your body and face? 1,2,3,4,5
22. Do you feel that you can't tolerate the cold in the winter than others? 1,2,3,4,5
23. Do you feel that you are more susceptible to catch a cold than others? 1,2,3,4,5
24. Do you sneeze even though you do not have a cold? 1,2,3,4,5
25. Do you often have a runny nose, even if it is not a cold? 1,2,3,4,5
26. Do you often have a stuffy nose, even if you are not a cold 1,2,3,4,5
27. Do you feel that though slightly active or inactive, you are easy to sweat (spontaneous)? 1,2,3,4,5
28. Do you have a sticky sweat ? 1,2,3,4,5
29. Do you find you have feet wet? 1,2,3,4,5
30. Are you easy to be allergies (drugs, food, smell, pollen)? 1,2,3,4,5
31. Is your skin easy to onset hives (urticaria and urticaria)? 1,2,3,4,5
32. Is your skin allergy to appeared purpura (purple petechia) 1,2,3,4,5?
33. Is your skin often appear black imperceptibly bleeding spots (subcutaneous hemorrhage)? 1,2,3,4,5
34. Is your skin susceptible to turn red and scratches after scratch? 1,2,3,4,5
35. Is your skin dryness? 1,2,3,4,5
36. Is your skin rough and delicate? 1,2,3,4,5
37. Do you feel pain here and there? 1,2,3,4,5
38. Is your face usually flushing or partial red? 1,2,3,4,5
39. Is your face or nose oily or greasy? 1,2,3,4,5
40. Is your complexion dullness? 1,2,3,4,5
41. Are you prone to acne? 1,2,3,4,5
42. Do you have a swollen eyes (the lower eyelids)? 1,2,3,4,5
43. have a dark circles around the eyes.? 1,2,3,4,5
44. Do you feel dryness of eyes? 1,2,3,4,5
45. Do you have eye congestion (redness) ? 1,2,3,4,5
46. Do you feel dry mouth and throat? 1,2,3,4,5
47. Do you have a feeling of blockage in the throat? 1,2,3,4,5
48. Do you feel a bitter taste in the mouth? 1,2,3,4,5
49. Do you have a sticky feeling in your mouth? 1,2,3,4,5
50. Do you sleep easily? 1,2,3,4,5
51. Do you have phlegm? 1,2,3,4,5
52. Do you feel uncomfortable after eating or drinking cool things? Or you donnot like to have cold things? 1,2,3,4,5
53. Do you sleep well 1,2,3,4,5
54. Do you easily sleep? 1,2,3,4,5
55. Are you susceptible to have loose stool? 1,2,3,4,5
56. Do you have a sticky stool? 1,2,3,4,5
57. Do you have a dry stool or constipation, 1,2,3,4,5
58. Do you have a lot of urine, or a frequent micturition? 1,2,3,4,5
59. Do you have a sense of fever in the urethra? Is your urine’s color darkness? 1,2,3,4,5
60. Do you have decadent leucorrhea? 1,2,3,4,5
